# Associations between Gilbert’s syndrome and personality characteristics

**DOI:** 10.47626/2237-6089-2020-0003

**Published:** 2021-03-18

**Authors:** Tolga Düzenli, Özgür Maden, Alpaslan Tanoğlu, Mustafa Kaplan, Yusuf Yazgan

**Affiliations:** 1 Department of GastroenterologyHitit University Erol OlcokTraining and Research HospitalCorumTurkey Department of Gastroenterology , Hitit University Erol Olcok Training and Research Hospital , Corum , Turkey .; 2 Department of PsychiatrySultan Abdulhamid Han Training and Research HospitalIstanbulTurkey Department of Psychiatry , Sultan Abdulhamid Han Training and Research Hospital , Istanbul , Turkey .; 3 Department of GastroenterologySultan Abdulhamid Han Training and Research HospitalIstanbulTurkey Department of Gastroenterology , Sultan Abdulhamid Han Training and Research Hospital , Istanbul , Turkey .; 4 Department of Internal MedicineSultan Abdulhamid Han Training and Research HospitalIstanbulTurkey Department of Internal Medicine , Sultan Abdulhamid Han Training and Research Hospital , Istanbul , Turkey .

**Keywords:** Gilbert’s syndrome, Temperament and Character İnventory, personality profile

## Abstract

**Objective:**

Gilbert’s syndrome (GS) is a benign genetic disorder that is characterized by intermittent mild jaundice in which the liver doesn’t process bilirubin properly. The aim of this study was to determine whether GS patients have a different personality structure and if there are associations between properties of temperament and character and total bilirubin levels.

**Methods:**

A total of 1665 young male individuals aged from 19 to 30 who were admitted for occupational examinations were included in this study. Careful patient history was taken, a detailed physical examination was conducted, and hematologic and biochemical tests and abdominal ultrasonography were performed. The Turkish version of the Temperament and Character Inventory (TCI) was administered to all participants. 81 patients diagnosed with GS and 150 randomly chosen healthy individuals (control group) were investigated with comparison and correlation analyses.

**Results:**

GS patients had higher scores than healthy controls for disorderliness (NS4) (p = 0.018), sentimentality (RD1) (p = 0.042), and fatigability (HA4) (p = 0.03). Moreover, Gilbert syndrome patients scored lower than controls for empathy (C2) (p = 0.041) and transpersonal identification (ST2) (p = 0.044). Bilirubin levels were positively associated with disorderliness (NS4) (r = 0.141, p = 0.032) and fatigability (HA4) (r = 0.14, p = 0.033).

**Conclusions:**

GS patients may have some different personality characteristics from healthy individuals. This study is an initial exploration of the personality structure of GS patients and the findings should be interpreted with caution. Further prospective studies are needed to identify the relationship between Gilbert disease and personality characteristics.

## Introduction

Gilbert’s syndrome (GS) is a benign genetic disorder that is characterized as intermittent mild jaundice, in which the liver doesn’t process bilirubin properly. The mechanism of GS is linked to reduced uridine diphosphate-glucuronyl-transferase (UGT) 1A1 activity, resulting in unconjugated hyperbilirubinemia. ^1^ Unconjugated hyperbilirubinemia occurs intermittently when patients are subject to stress factors, such as physical stress, prolonged fasting, and/or poor diet. ^1^ Emotional stimuli are associated with increases in the oxidative metabolites of bilirubin in human urine. ^2^ On the other hand, GS is believed to reduce the risk of various diseases because of the antioxidant properties of bilirubin. ^3^

Although associations between emotional stimuli and increased oxidative metabolites of bilirubin in human urine have been demonstrated, it is not obvious whether GS patients have a different personality structure or whether there are associations between properties of temperament and character and total bilirubin levels.

The dimensional method has utility for personality assessment according to Cloninger’s psychobiological model. Personality has been modeled as two discrete components: character and temperament. Temperament is defined as a constitutionally or biologically based segment of the personality, expresses the automatic sentimental reaction to events, and is influenced by emotional, motor, and attentional reactivity and self-regulation, underlying a variety of personal decisions. ^4 , 5^

Character is defined as a self-concept, and is influenced by our interactions with people and our experiences and facility for learning. Therefore, these interactions and experiences enable greater flexibility and thus organization of personal differences in values and intentions.

Cloninger hypothesized that neurotransmitters were related to behavioral manifestations. ^4^ In this context, as an inherited disorder, GS could be associated with altered glucuronidation rates of these metabolites and, consequently, with behavioral manifestations.

The aim of this study was to investigate associations with temperament and character properties in GS patients and healthy individuals.

## Methods

### Study design and participants

A total of 1665 young male individuals aged from 19 to 30 who were admitted to our hospital for occupational examinations were included in this retrospective, cross-sectional study. The study was approved by the institutional ethics committee and was therefore performed in accordance with the ethical standards laid down in the 1964 Helsinki Declaration and its later amendments. The patients included in the study provided written consent. Patient history was taken with care, a detailed physical examination was conducted, and hematologic and biochemical tests and abdominal ultrasonography were performed. All participants were examined by the departments of internal medicine, general surgery, cardiology, otorhinolaryngology, dermatology, ophthalmology, orthopedics, neurology, and psychiatry. After 12-hour overnight fasting, blood samples were obtained from patients’ brachial veins in the biochemistry department and tested in approximately 1-2 hours.

In addition to total and direct/indirect bilirubin levels; hemogram, alanine aminotransferase (ALT), aspartate aminotransferase (AST), alkaline phosphatase (ALP), gamma glutamyl transferase (GGT), lactate dehydrogenase (LDH), glucose, urea, creatinine, sodium, potassium, lipid panel (HDL-cholesterol, LDL-cholesterol, total cholesterol, triglyceride), sedimentation rate, thyroid stimulating hormone (TSH), vitamin b12, folate, ferritin, albumin, and hepatitis markers (HbsAg, anti-HCV, anti-HIV) were measured.

**Figure 1:**
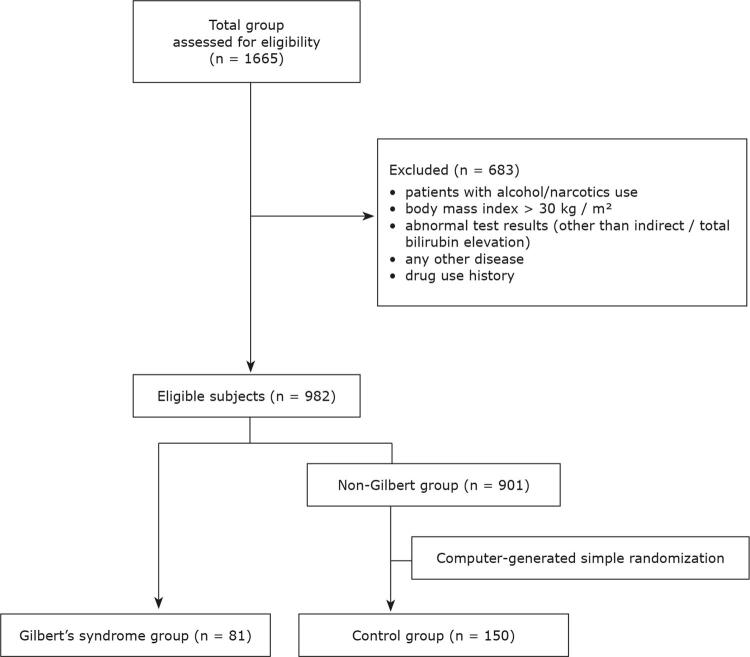
Patient enrollment and classification.

Patients with alcohol/narcotics use, body mass index > 30 kg/m ^2^ , abnormal test results (other than indirect/total bilirubin elevation), or any known history of disease or drug use were excluded from the study (n = 683). A total of 982 people were eligible according to these criteria ( Figure 1 ). Of these, 81 patients who had a total bilirubin value of 1.4 mg/dL or more, with no evidence of hemolysis or other pathological results (all of the tests mentioned above, including normal hemogram parameters, normal cholestatic enzymes, negative serological markers of viral hepatitis, and normal abdominal ultrasonography) were diagnosed as having GS. ^1 , 6^ A control group was formed by randomly selecting 150 patients from the remaining 901 patients. These two groups were investigated by comparison and correlation analyses.

### Personality characteristics

All study participants were instructed to fill in the Turkish version of the TCI for personality assessment. ^4 , 5 , 7^ Temperament is divided into four different independent parts in this representation, as follows: novelty seeking (NS), harm avoidance (HA), reward dependence (RD), and persistence (PS). In turn, NS consists of exploratory excitability (NS1), impulsiveness (NS2), extravagance (NS3), and disorderliness (NS4); HA contains the elements anticipatory worry (HA1), fear of uncertainty (HA2), shyness (HA3), and fatigability (HA4); RD comprises sentimentality (RD1), openness to friendly communication (RD2), attachment (RD3), and dependence (RD4); and PS is a fourth independent dimension.

Character is made up of three dimensions; cooperativeness (C), self-directedness (SD), and self-transcendence (ST). In turn, C includes social acceptance (C1), empathy (C2), helpfulness (C3), compassion (C4), and pure-hearted conscience (C5); SD contains responsibility (SD1), purposefulness (SD2), resourcefulness (SD3), self-acceptance (SD4), and congruence (SD5); and ST is made up of self-forgetfulness (ST1), transpersonal identification (ST2), and spiritual acceptance (ST3).

### Statistical analyses

The Statistical Package for the Social Sciences (SPSS) version 15.0 was used for statistical analysis. Results with p < 0.05 were considered statistically significant. Descriptive properties of the study groups were expressed as mean ± standard deviation (SD) or percentage (%) and number (n). The means of the measurements for each patient were calculated and analyzed. The Kolmogorov-Smirnov test was used to determine conformity to normal distribution. The Mann Whitney U test was used to compare variables which did not fit a normal distribution. We used chi-square tests to compare qualitative data. Pearson and Spearman tests were used for correlation analyses.

## Results

There were 81 patients in the GS group and 150 patients in the control group. All of the patients were male. The mean age was 22.7±1.6 years in the GS group and 23.1±1.6 years in the control groups (p > 0.05). There were no differences between the two groups in terms of the following demographic variables: marital status, number of siblings or their order, where or with whom they were living, previous job experience, economic and family status, mothers’ and fathers’ health status or educational levels, or academic and school performance ( Table 1 ).

**Table 1:** Sociodemographic characteristics of Gilbert’s syndrome patients and control subjects

	Gilbert (n = 81)	Control (n = 150)	P
Age, mean	22.7 *±* 1.6	23.1±1.6	0.055
Marital status			
Married	0	3.3	0.165
Single	100	96.7	
Siblings			
Number	2.4±1.4	2.3±1.3	0.856
Order	1.9±1.1	2.0±1.0	0.384
Residence			
Village	8.6	14.7	0.252
Town	18.5	12	
City	42	48	
Metropolis	30.9	25.3	
Living arrangements			
Family	37.0	35.3	0.392
Friend	27.2	36.0	
Alone	13.6	8.0	
Youth hostel	22.2	20.7	
Job experience			
Yes	44.4	40	0.513
No	55.6	60	
Economic status			
Low	16.0	18.0	0.912
Medium	81.5	80.0	
High	2.5	2.0	
Family status			
Nuclear	79.0	78.7	0.994
Extended	17.3	17.3	
Other*	3.7	4.0	
Mother-ex/alive			
Deceased	1.2	2	0.562
Alive	98.8	98	
Father-ex/alive			
Deceased	3.7	6.7	0.551
Alive	96.3	93.3	
Education-mother			
Uneducated	12.3	5.3	0.302
Elementary	58.0	64.7	
Middle school	16.0	16.7	
High school	12.3	10.0	
College	1.2	3.3	
Education-father			
Uneducated	1.2	1.3	0.176
Elementary	50.6	44.0	
Middle school	25.9	18.7	
High school	11.1	24.0	
College	11.1	12.0	
Family income			
Lower	16.0	12.7	0.114
Middle	81.5	87.3	
Upper	2.5	0	
Academic/school performance			
Very good	13.6	8.0	0.546
Good	54.3	61.3	
Not bad	30.9	29.3	
Bad	1.2	1.3	

Data presented as percentages, unless otherwise specified. *Including stepfamilies; single-parent families, and other configurations not classified as nuclear or extended families.

The GS patients had significantly higher scores than the controls for disorderliness (NS4) (p = 0.018), sentimentality (RD1) (p = 0.042), and fatigability (HA4) (p = 0.03) ( Table 2 ). GS patients scored lower than controls for the empathy (C2) (p = 0.041) and transpersonal identification (ST2) dimensions (p = 0.044). There were no statistically significant differences between the two groups for any of the other parameters. The mean scores of the TCI dimensions for GS patients and control subjects are presented in Table 2 .

**Table 2:** Mean TCI dimension scores for Gilbert’s syndrome patients and control subjects

	Gilbert (n = 81)	Control (n = 150)	p
Total novelty seeking (NS) score	15.6±3.0	15.2±3.2	0.204
Exploratory excitability (NS1)	7.1±1.5	7.1±1.5	0.944
Impulsiveness (NS2)	2.2±1.4	2.3±1.5	0.900
Extravagance (NS3)	3.5±1.3	3.4±1.4	0.463
Disorderliness (NS4)	2.8±1.4	2.3±1.4	0.018*
Total harm avoidance (HA) score	7.7±4.3	7.2±4.0	0.409
Anticipatory worry (HA1)	2.7±1.8	2.6±1.8	0.541
Fear of uncertainty (HA2)	2.4±1.3	2.3±1.4	0.751
Shyness (HA3)	1.0±1.3	1.1±1.4	0.466
Fatigability (HA4)	1.6±1.3	1.3±1.4	0.030*
Total reward dependence (RD) score	14.8±3.3	14.7±2.7	0.901
Sentimentality (RD1)	6.4±1.6	5.9±1.5	0.042*
Attachment (RD3)	5.3±1.7	5.6±1.5	0.141
Dependence (RD4)	3.1±1.3	3.2±1.4	0.476
Persistence score	6.7±1.2	6.4±1.2	0.551
Total self-directedness (SD) score	34.0±5.0	34.3±4.7	0.610
Responsibility (SD1)	6.3±1.5	6.6±1.4	0.267
Purposefulness (SD2)	6.3±0.9	6.4±1.0	0.590
Resourcefulness (SD3)	4.2±0.9	4.2±1.0	0.464
Self-acceptance (SD4)	6.7±2.4	6.9±2.2	0.821
Congruence (SD5)	10.4±1.4	10.3±1.4	0.511
Total cooperativeness (C) score	35.0±3.9	35.4±4.2	0.313
Social acceptance (C1)	7.2±1.0	7.3±1.0	0.416
Empathy (C2)	5.4±1.1	5.7±1.2	0.041*
Helpfulness (C3)	5.8±1.4	5.8±1.3	0.897
Compassion (C4)	8.9±1.4	9.0±1.3	0.674
Pure-hearted conscience (C5)	7.6±1.1	7.5±1.1	0.548
Total self-transcendence (ST) score	17.5±4.8	18.3±5.2	0.385
Self-forgetfulness (ST1)	4.7±1.7	4.9±1.9	0.550
Transpersonal identification (ST2)	6.0±1.9	6.5±1.9	0.044*
Spiritual acceptance (ST3)	6.7±2.8	6.9±2.8	0.800

TCI = Temperament and Character Inventory.

* p < 0.05 is considered statistically significant.

We also applied an effect size (ES) measure to the significance of the statistical differences. ES indicates that if two groups’ means do not differ by at least 0.2 standard deviations, the difference is trivial, even if it is statistically significant. Our study groups’ results are presented in Table 3 .

**Table 3:** Associations between TCI and bilirubin levels

	Gilbert (n = 81)	Control (n = 150)	p	Effect size (Cohen’s d)
Disorderliness (NS4)	2.8±1.4	2.3±1.4	0.018*	0.4
Fatigability (HA4)	1.6±1.3	1.3±1.4	0.030*	0.2
Sentimentality (RD1)	6.4±1.6	5.9±1.5	0.042*	0.4
Empathy (C2)	5.4±1.1	5.7±1.2	0.041*	0.3
Transpersonal identification (ST2)	6.0±1.9	6.5±1.9	0.044*	0.3

TCI = Temperament and Character Inventory.

d = 0.2 is considered a ‘small’ effect size, 0.5 represents a ‘medium’ effect size, and 0.8 is a ‘large’ effect size.

There were low-positive significant correlations between total bilirubin levels and disorderliness (NS4) (r = 0.141, p = 0.032) and fatigability (HA4) (r = 0.14, p = 0.033) ( Table 4 ). There were no significant correlations between the other TCI dimensions and patients’ total bilirubin levels.

**Table 4:** Associations between TCI and total bilirubin levels

Variable	r	P	Variable	r	p	Variable	r	p
NS total	0.111	0.092	RD total	-0.008	0.901	C total	-0.058	0.383
NS1	0.028	0.669	RD1	0.112	0.090	C1	-0.057	0.385
NS2	0.000	0.998	RD3	-0.074	0.264	C2	-0.116	0.077
NS3	0.072	0.273	RD4	0.063	0.339	C3	-0.009	0.887
NS4	0.141	0.032*				C4	0.029	0.661
						C5	-0.001	0.985
HA total	0.014	0.832	SD total	-0.015	0.826	ST total	-0.023	0.723
HA1	0.024	0.718	SD1	-0.087	0.186	ST1	0.013	0.840
HA2	-0.018	0.787	SD2	0.016	0.811	ST2	-0.088	0.181
HA3	-0.073	0.271	SD3	0.008	0.901	ST3	-0.011	0.864
HA4	0.140	0.033*	SD4	-0.038	0.569			
			SD5	0.083	0.211			
P	0.000	0.996						

*p < 0.05 is considered statistically significant.

NS = novelty seeking; NS1= exploratory excitability; NS2 = impulsiveness; NS3 = extravagance; NS4 = disorderliness; HA = harm avoidance; HA1 = anticipatory worry; HA2 = fear of uncertainty; HA3 = shyness; HA4 = fatigability; RD = reward dependence; RD1 = sentimentality; RD3 = attachment; RD4 = dependence; P = persistence; SD = self-directedness; SD1 = responsibility; SD2 = purposefulness; SD3 = resourcefulness; SD4 = self-acceptance; SD5 = congruence; C = cooperativeness; C1 = social acceptance; C2 = empathy; C3 = helpfulness; C4 = compassion; C5 = pure-hearted conscience; ST = self-transcendence; ST1 = self-forgetfulness; ST2 = transpersonal identification; ST3 = spiritual acceptance.

## Discussion

The Temperament and Character Inventory-240 (TCI-240) has not been used with GS patients up to the present. Our current study showed that GS patients have a characteristic personality profile with higher scores for disorderliness, sentimentality, and fatigability and lower scores for empathy and transpersonal identification than healthy individuals.

Stress factors are associated with increased bilirubin and its metabolites in urine. Urinary excretion of bilirubin oxidative metabolites increases in septic patients. ^8^ Novío et al. ^9^ reported that stressed mice had higher oxidative metabolites of bilirubin than non-stressed mice and observed that levels declined after alprazolam doses were given to the stressed animals.

On the other hand, bilirubin has important antioxidant properties, which has been confirmed over the last 2 decades, contributing to defense against increased oxidative stress. Experimental and also clinical studies have indicated associations between low bilirubin concentrations and cardiovascular diseases, diabetes mellitus, certain cancers, autoimmune diseases such as lupus erythematosus or rheumatoid arthritis, and psychiatric disorders such as schizofrenia. ^7^ Subjects with mildly elevated blood bilirubin levels, typical of GS, have decreased risk of these diseases. ^10^ Maruhashi et al. ^11^ evaluated the role of oxidative stress in endothelial function in patients with GS under normal conditions without cardiovascular risk factors. Patients with GS had low levels of oxidative stress associated with hyperbilirubinemia and enhanced endothelium-dependent vasodilation. ^11^

The relationship between bilirubin levels and psychopathology has been investigated in a limited number of studies. Yamaguchi et al. ^2^ indicated that emotional stimuli are associated with an increase in oxidative metabolites of bilirubin in human urine. Miyaoka et al. ^12^ reported that bilirubin oxidative metabolites (biopyrrins) are increased in urine from patients with psychiatric disorders (schizophrenia and depression). Bilirubin concentration also exhibited correlation with positive symptoms in patients with schizophrenia. ^13^ Tang et al. ^14^ demonstrated that high bilirubin level is associated with post-stroke depression. These findings suggest that behavioral states are associated with increases in the oxidative metabolites of bilirubin in human urine. Less clear and still under study is the possible relation between serum bilirubin levels and neurological diseases. ^15^ Kao et al. ^16^ investigated the relationship between serum total bilirubin levels and functional dependence in older adults, revealing that higher serum total bilirubin levels were associated with lower likelihood of functional dependence. Oren et al. ^17^ reported that nocturnal bilirubin levels were lower in seasonal depression patients than in controls. However, notwithstanding these neurochemical mechanisms, the social repercussions of a possible clinical manifestation with GS should not be overlooked. For example, repercussions of the stigma caused by chronic/intermittent jaundice of individuals with GS may also have an effect on personality characteristics.

With regard to temperament, individuals who score low on the disorderliness subscale have a tendency to be disorganized, disordered, chaotic, and unsystematic. They do not like activities with strict rules and regulations. ^4 , 5^

High harm avoidance scores are related to depression and anxiety symptoms, but there was no difference in harm avoidance parameters except fatigability. Although there is no consensus on fatigue as a symptom of GS, higher fatigability may be an expected outcome for GS patients. ^18 , 19^ Moreover, with a significant low correlation between serum bilirubin levels and fatigability, our study may have revealed new evidence on the subject of fatigability for GS patients.

Reward dependence reflects behavior response, which stimulates a social reward and reflects a genetic tendency that stimulates continuation of this behaviour. ^4 , 5^ In the present study, scores for the reward dependence subscale sentimentality were higher among patients with GS than in the control group. Significantly higher sentimentality scores in patients with GS may be due to placing more importance on social consent and to being susceptible or easily impressionable by others.

Character consists of three parts; cooperativeness (C), self-directedness (SD), and self-transcendence (ST). Within these, our study demonstrated lower scores for empathy and transpersonal identification in GS patients. Empathy is described as a sensation of unity or identification with other individuals and is said to enable improved communication and compassion for others. ^4 , 5^ Transpersonal identification is related to spiritual acceptance or to apprehension of relationships that cannot be explained by analytical reasoning or demonstrated to others by objective observations. ^4 , 5^

Cloninger hypothesized that neurotransmitters were related with behavioral manifestations, such as, for example, serotonin with harm avoidance (behavioral inhibition); norepinephrine with reward dependence (behavioral maintenance); dopamine with novelty seeking (behavioral activation), and glutamine with persistence (behavioral perseverance). ^4 , 5^ In this context, as an inherited disorder, GS may be associated with altered glucuronidation rates of these metabolites. Lee et al. ^20^ proposed that in a subgroup of Gilbert’s syndrome patients, homozygosity for dual UGT 1A1 and UGT 1A6 polymorphisms may lead to altered metabolism and elimination of serotonin. Peters et al. ^21^ mentioned that UGTs play important roles in mediating biological activity of endogenous substrates in addition to their roles in drug distribution, metabolism, and excretion processes.

This study can be considered an initial exploration of the personality structure of GS patients. However, we believe that clinicians should be cautious about interpreting the findings, in view of the study’s confounding factors and limitations. Firstly, the size of the sample was relatively small for extrapolation of results. More studies with different psychological testing methods could be used to generalize our findings. Secondly, our study was a retrospective cross-sectional study. Further prospective studies are required to identify the causality of the links between Gilbert’s disease and personality characteristics. Thirdly, the TCI is a self-report questionnaire and may be influenced by environmental factors. Fourthly, all the subjects were male and young, but this may also have ensured greater homogeneity of the groups and exclusion of age and gender effects on TCI results. Additionally, GS was diagnosed from clinical and laboratory parameters in this study, whereas definitive diagnosis can be made by genetic mutation analysis. Finally, the detailed results of medical departments’ clinical and psychiatric evaluations were not available, only their final assessments and decisions, which could constitute a potential confounding factor between groups. Nevertheless, the study also included subjects who were assessed as ‘healthy’ by each of the departments. Subjects with any pathological conditions were excluded from the study.

In conclusion, our findings demonstrate significant differences in personality features (TCI 240) between GS patients and healthy individuals. There might be a relationship between GS and personality characteristics and therefore patients’ personality features might merit attention when evaluating patients with GS. On the other hand, our findings were statistically significant, but the effect size was small and there are some confounding factors and limitations. Further prospective studies are needed to identify the relationship between Gilbert’s disease and personality characteristics.
